# Comparison of Periprosthetic Femoral Bone Remodeling After Total Hip Arthroplasty with Zimmer VerSys Taper Stem or VerSys Midcoat Stem: Long-Term Outcomes

**DOI:** 10.3390/jcm15135181

**Published:** 2026-07-02

**Authors:** Keishi Kimura, Norio Imai, Yuki Hirano, Yoji Horigome, Hiroyuki Kawashima

**Affiliations:** 1 Department of Regenerative and Transplant Medicine, Division of Orthopedic Surgery, Niigata University Graduate School of Medicine, Dentistry and Health Sciences, Niigata 951-8510, Japan; 2 Division of Comprehensive Musculoskeletal Medicine, Niigata University Graduate School of Medicine, Dentistry and Health Sciences, Niigata 951-8510, Japan

**Keywords:** total hip arthroplasty, stress shielding, cortical hypertrophy, bone remodeling

## Abstract

**Background/Objectives**: We compared long-term radiological femoral bone remodeling after total hip arthroplasty (THA) with either the VerSys Taper stem (T) or VerSys Midcoat stem (M). **Methods:** Of 206 patients who underwent unilateral primary THA, 157 (T group: 65; M group: 92) were followed up for >15 years. Stress shielding was evaluated according to Engh’s classification, cancellous condensation and cortical hypertrophy in each zone were assessed according to Gruen’s zone, and fixation was evaluated using Engh’s classification. We investigated the grade of each stem at each point in stress shielding. **Results:** Grade 3 or 4 stress shielding incidence was higher in the T group than in the M group, and stress shielding grades at each time point were significantly higher in the T group. The stress shielding grade showed progressively greater divergence in the T group, consistent with a significant stem × time interaction. The generalized linear mixed model showed significant zonal variation in cancellous condensation but no effects of stem type or stem × zone interaction. Similarly, cortical hypertrophy showed no significant effects of stem type, zone, or their interaction. There were no cases of unstable fixation in either group; however, 90.2% of patients in the M group had bone ingrowth, whereas 23.1% of patients in the T group had fibrous stability. There were no cases of revision owing to aseptic loosening in either group. **Conclusions:** These findings suggest that the primary long-term difference between the stem designs lies in the trajectory of stress-shielding progression rather than in zonal bone responses.

## 1. Introduction

Total hip arthroplasty (THA) is a widely performed surgical intervention that significantly improves pain, mobility, and overall quality of life for patients suffering from degenerative hip disorders, such as osteoarthritis, rheumatoid arthritis, or avascular necrosis [1,2,3]. With the increasing demand for joint replacements and the expansion of indications to younger and more active populations, long-term implant performance and bone preservation have become critical issues in contemporary orthopedic practice [4,5,6].

Cementless femoral stems have been particularly favored for their potential to achieve durable biological fixation, avoiding the complications related to cement degradation or loosening [7,8,9]. To this end, manufacturers have developed a range of stem designs with varying geometries and surface treatments aimed at promoting osseointegration and optimizing load transfer [10,11,12,13,14]. The VerSys Hip System (Zimmer Biomet) incorporates anatomical data derived from cadaveric studies to provide stems that accommodate different femoral morphologies [14]. It offers two uncemented stem designs: the VerSys Taper stem and the VerSys Midcoat stem.

Both stem types share a similar proximal geometry, including a porous fiber metal coating made of commercially pure titanium, further treated with a hydroxyapatite/tricalcium phosphate (HA/TCP) coating to facilitate bone ingrowth. Additionally, a corundumized surface is applied distal to the proximal coating, and in the Taper stem this coating extends more distally than in the Midcoat stem, which enhances osseointegration by combining osteoconductivity with controlled resorption [15,16,17,18]. However, distal geometries differ significantly; the Taper stem features a tapered, polished design intended to limit distal fixation, whereas the Midcoat stem has a fluted distal section aimed at improving rotational stability and minimizing stress transfer to the proximal femur [19,20,21]. Therefore, the observed differences in radiographic findings and remodeling patterns are more likely attributable to differences in the distal design rather than differences in the osseointegration potential.

Despite their widespread use in the early 2000s, the application of these stems has gradually declined with the emergence of new short stems and tapered wedge designs [22,23,24]. Although several studies have reported favorable midterm outcomes using the VerSys system [25,26], data on long-term periprosthetic bone remodeling remain limited. These conventional stems have been used in a large number of case over a long period, and many patients still have the implant inside their bodies. Therefore, it is important to clarify their long-term safety, fixation, and remodeling characteristics. Moreover, the selection of femoral stem design must consider not only short-term fixation stability but also the long-term effects on bone remodeling and the potential need for future revision surgery. Given the critical role of stress shielding and cortical hypertrophy in the progression of implant-related complications, such as periprosthetic fractures or implant loosening, it is vital to assess how these two stem designs influence femoral bone adaptation over time.

Stress shielding contributes to concentrated forces in the diaphyseal region, reducing physiological loading in the proximal femur. This can lead to bone resorption and weakening of the proximal femoral structure, which is clinically relevant because stress shielding is associated with an increased risk of periprosthetic fractures, particularly in older patients and those with osteoporosis.

Cortical hypertrophy is a localized bone formation response to repetitive distal stress and reflects increased mechanical loading on the cortical bone. Occasionally, this can cause discomfort or thigh pain.

We hypothesized that because the grit-blast surface treatment is applied more distally on Taper stems than on Midcoat stems, Taper stems would achieve fixation at a more distal location. Consequently, this would result in more pronounced stress shielding proximally and in distal cortical hypertrophy. Therefore, this retrospective study aimed to compare long-term radiographic outcomes, specifically femoral bone remodeling patterns, between the VerSys Taper and Midcoat stems over a minimum 15-year follow-up period. We aimed to provide new insights into the long-term implications of stem design on femoral remodeling and implant stability.

## 2. Materials and Methods

This retrospective cohort study was conducted in accordance with the ethical principles outlined in the Declaration of Helsinki and approved by the Institutional Review Board of Niigata University (Approval No. 2023-0138). This study was registered on 4 August 2021. Given the retrospective nature of the study and the use of de-identified data, the requirement for written informed consent was waived. An opt-out approach was applied, and no patients requested exclusion.

We initially reviewed 206 patients who underwent unilateral primary THA using the same surgical approach (anterolateral supine approach) and standardized preoperative and postoperative rehabilitation protocols at our institution between 1 October 2002, and 30 September 2007. No major intraoperative complications, including femoral fracture, nerve injury, or vascular injury, were recorded in the reviewed medical records. All patients underwent standardized postoperative rehabilitation beginning on the first postoperative day, including range-of-motion exercises, muscle strengthening, and gait training without limitation of weight-bearing according to institutional protocols.

**Figure 1:**
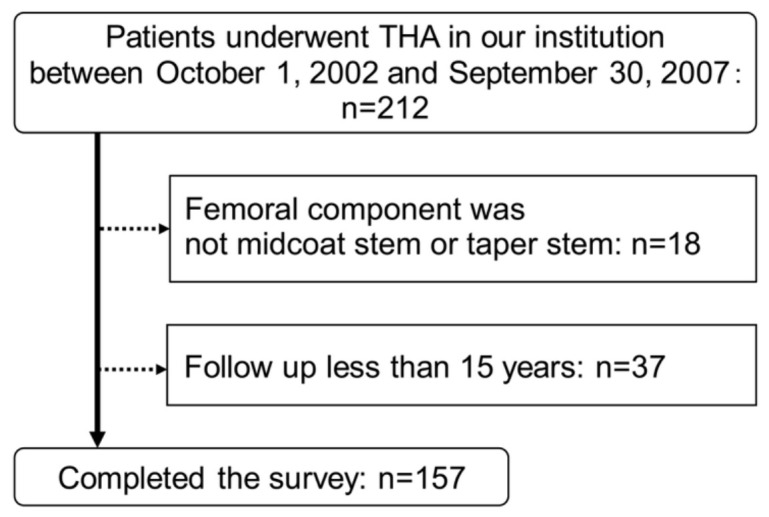
Flowchart of the participants of this survey.

**Figure 2:**
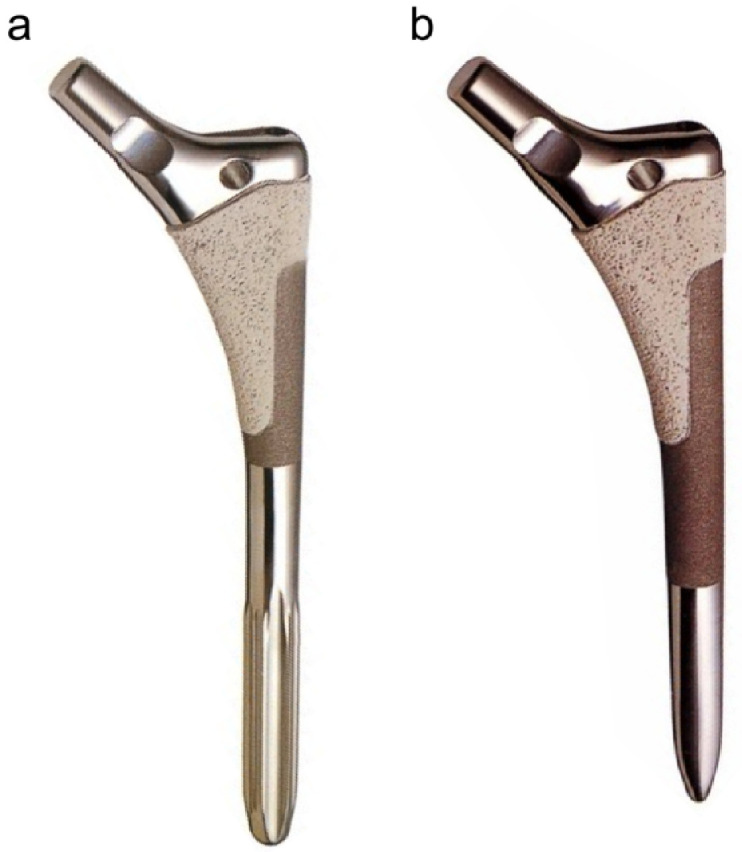
VerSys hip system. (**a**) VerSys Midcoat stem (**b**) VerSys Taper stem. The proximal body is coated with commercially pure titanium fiber metal and HA/TCP. The distal shape differs in that the Taper stem has a tapered shape, while the Midcoat stem is fluted. Zimmer Biomet granted permission for the use of the images in this publication.

Of these, 157 patients (32 males and 125 females) with radiographic follow-up exceeding 15 years were included in the final analysis. The patients were divided into two groups based on the type of femoral stem used: the Midcoat group (M group, n = 92) and Taper group (T group, n = 65). The patient selection flowchart is shown in Figure 1, and stem morphologies are shown in Figure 2.

### 2.1. Radiographic Assessment

Radiological evaluations were conducted at multiple postoperative time points (1, 3, 5, 10, and 15 years) as well as at the latest follow-up visit. All radiographs were obtained in standardized anteroposterior and lateral views, centered on the hip joint. No patients were excluded based on imaging conditions.

Stress shielding was graded using Engh’s classification, which categorizes bone resorption due to altered stress distribution into four levels (Grade 0 to Grade 4) [27]. Stress shielding was evaluated using Gruen zones 1–7 on anteroposterior radiographs, allowing for a detailed evaluation of both proximal and distal femoral remodeling.

Cancellous condensation and cortical hypertrophy were radiographically assessed using Gruen’s zone classification [28]. Cancellous condensation was defined as an area of increased cancellous bone density adjacent to the stem compared with the immediate postoperative radiograph, and cortical hypertrophy was judged as present when cortical thickening was clearly visible on subsequent radiographs. These radiographic signs represent localized bone responses to implant loading, where cancellous condensation reflects endosteal bone apposition and cortical hypertrophy indicates thickening of the cortical bone due to mechanical stress. Both cancellous condensation and cortical hypertrophy were recorded as present or absent in each zone (zones 1–7).

Fixation stability of the femoral stem was evaluated based on the radiographic criteria proposed by Engh et al. [29], classifying fixation into one of three categories: bone ingrowth (stable osseointegration), fibrous stability (micromotion with fibrous encapsulation), and unstable fixation (radiolucent lines and implant migration). Only radiographs with clear visibility of the cortical boundaries and implant–bone interfaces were used for assessment. No patients were excluded because of poor image quality.

Two orthopedic surgeons independently evaluated all radiographic images, and discrepancies were resolved through consensus.

Radiographs were evaluated as a chronological series for each patient to allow for assessment of temporal changes in bone remodeling. Complete blinding was not feasible because the stem designs were radiographically distinguishable, and the raters were also aware of the follow-up duration. Intraobserver and interobserver reliabilities were calculated using intraclass correlation coefficients (ICCs) and a two-sided 95% confidence interval (CI). Intraobserver reliability was assessed by the same observer performing two measurements at intervals of at least one week. Interobserver reliability was evaluated by comparing the measurements obtained by a second observer with those by the primary observer. Statistical significance was set at *p* < 0.01. The intraobserver reliability was ICC(3,1) = 0.974 (95% CI: 0.959–0.983), and the interobserver reliability was ICC(2,1) = 0.949 (95% CI: 0.921–0.966).

### 2.2. Statistical Analysis

For demographic data of the participants, statistical analyses were performed using IBM SPSS Statistics version 28.0 (IBM Corp., Armonk, NY, USA). Statistical significance was set at *p* < 0.05. Unpaired *t*-tests were used to compare continuous variables, such as age, radiographic anteversion, inclination angles, and stem version. Categorical variables, including sex, surgical side, and diagnosis, were compared using the chi-square test or Fisher’s exact test depending on the cell size in the contingency tables.

For the radiological evaluations, statistical analyses were conducted using jamovi (version 2.6.44) and the GAMLj3 module. Stress shielding grades (0–4) were analyzed longitudinally using linear mixed-effects models, with stem type, time point, and their interaction as fixed effects and patient ID specified as a random intercept to account for within-subject correlation across repeated measurements. Time was treated as a categorical factor.

Cancellous condensation and cortical hypertrophy were recorded as binary outcomes (present/absent) in each Gruen zone and analyzed using generalized linear mixed models with a binomial distribution and logit link function. Stem type, zone, and their interactions were included as fixed effects, with patient ID specified as a random intercept to account for the clustering of zones within individuals.

Estimated marginal means with 95% CIs were calculated to facilitate interpretation of significant effects. A post hoc power analysis was not performed because mixed-effects modeling was used to appropriately account for the hierarchical data structure.

## 3. Results

The demographic and clinical characteristics of the 157 participants included in the study are summarized in Table 1. The average follow-up was 18.4 years (range: 15–22 years), allowing for a robust long-term analysis. No statistically significant differences were observed between the T and M groups in terms of age at surgery, sex distribution, operative side, primary diagnosis (e.g., osteoarthritis and osteonecrosis), surgical approach, or radiographic parameters such as stem anteversion, cup inclination, or anteversion angles. These findings confirmed that the two groups were well-matched, minimizing potential confounding due to baseline variables.

**Figure 3:**
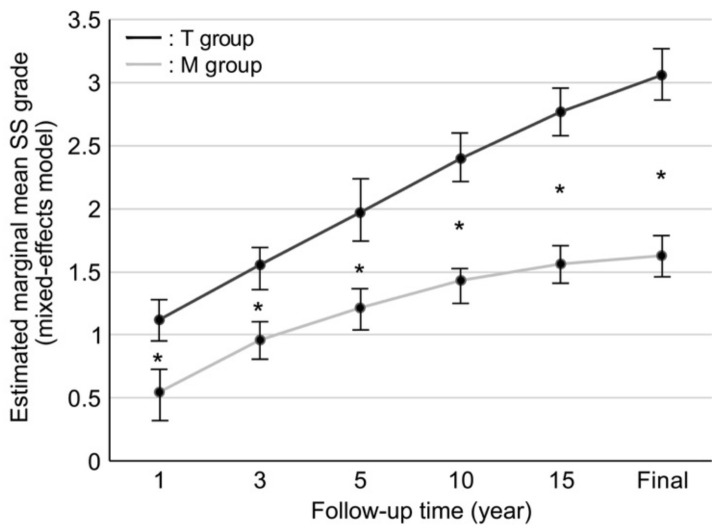
Longitudinal progression of stress shielding grades. Values represent the estimated marginal means derived from a linear mixed-effects model, including stem type, time, and their interaction, with the patient ID specified as a random intercept. Error bars indicate 95% confidence intervals (* *p* < 0.001). SS, stress shielding.

The incidence of severe stress shielding, defined as grade 3 or 4 according to Engh’s classification, was notably higher in the T group (64.6%) than in the M group (43.4%) at the final follow-up, as shown in Table 2. A linear mixed-effects model demonstrated significant main effects of stem type and time (both *p* < 0.001) as well as a significant stem × time interaction (*p* < 0.001), indicating that the longitudinal progression of stress shielding differed between the two stem designs. The estimated marginal means showed that at 5 years postoperatively, the mean stress shielding grade was 1.22 in the M group and 1.97 in the T group. This difference further increased at 10 years (1.44 vs. 2.40) and 15 years (1.56 vs. 2.77), demonstrating a progressively greater divergence over time in the T group (Figure 3).

This gradual escalation of stress shielding severity in the T group suggests a biomechanical consequence of the distal load transfer, which may suppress physiological stress in the proximal femur. The more proximally concentrated stress shielding in the M group implies that this stem may better preserve the proximal bone stock.

**Figure 4:**
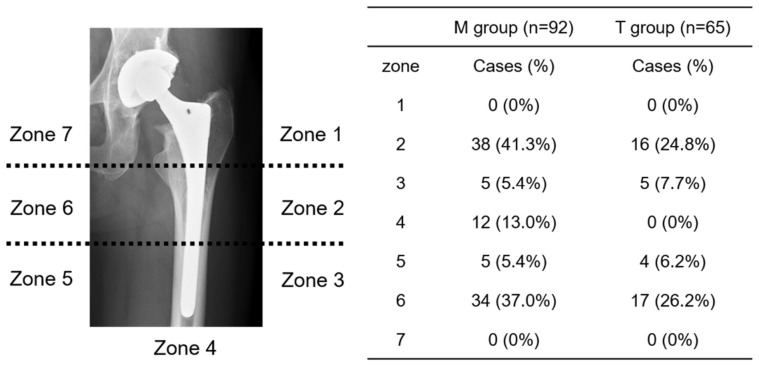
Cancellous condensation. Cancellous condensation was most common in Gruen’s zones 2 and 6 in both groups (M/T: 78%/51%), while cancellous condensation in zones 3 and 5 was more common in the T group (M/T: 11%/14%).

Cancellous condensation was frequently observed in Gruen zones 2 and 6 in both groups, with a markedly higher frequency in the M group (78%) than the T group (51%). These zones correspond to the metaphyseal regions that typically receive load transfer from proximally fixed stems. Interestingly, in the distal zones (Gruen zones 3 and 5), the T group exhibited a slightly higher incidence of cancellous condensation than the M group (14% vs. 10%, respectively), suggesting a tendency toward distal stress transfer with the tapered stem design. These findings are presented in Figure 4.

Cancellous condensation is considered a radiographic marker of localized osteogenesis in response to mechanical stimulation. The presence of cancellous condensation in distal regions may indicate that the Taper stem engages the cortical bone further down the femoral shaft than the Midcoat stem.

**Figure 5:**
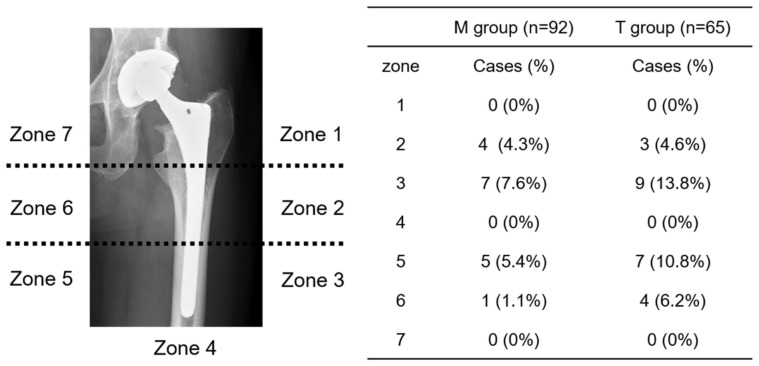
Cortical hypertrophy. Cortical hypertrophy occurred more often in the T than the M group (M/T: 16%/25%) and in zones 3 and 5 (M/T: 13%/25%).

Cortical hypertrophy was noted more frequently in the T group (25%) than in the M group (16%), particularly in Gruen zones 3 and 5 (T: 25%, M: 13%), as shown in Figure 5. Cortical hypertrophy represents thickening of the cortical bone in response to an increased mechanical load. The localized distribution of cortical hypertrophy in the distal zones further supports the hypothesis that the Taper stem transmits load to a more distal region, in contrast to the more proximal loading pattern observed with the Midcoat stem.

In the generalized linear mixed model, a significant main effect of zone was observed (*p* < 0.001), indicating a regional variation in cancellous condensation. However, no significant main effects of stem type (*p* = 0.894) or stem × zone interaction (*p* = 0.778) were detected.

For cortical hypertrophy, no significant effects of stem type (*p* = 0.990) or zone (*p* = 0.213) were observed, and the stem × zone interaction was not significant (*p* = 0.938), suggesting no stem-dependent differences in zonal distribution when accounting for within-patient correlations.

Radiographic assessment of femoral stem fixation revealed no cases of unstable fixation in either group according to Engh’s classification. However, there were marked differences in the mode of fixation between the groups; bone ingrowth stability was achieved in 90.2% of cases in the M group. In contrast, only 76.9% of the patients in the T group achieved bone ingrowth, with 23.1% exhibiting fibrous stability.

These data are presented in Table 3. There was no requirement for revision surgery due to aseptic loosening in either group, indicating satisfactory long-term clinical performance.

## 4. Discussion

This study evaluated the long-term femoral bone remodeling following THA using two cementless stem designs over a follow-up period exceeding 15 years. By applying mixed-effects modeling to appropriately account for the hierarchical and longitudinal structures of the data, we demonstrated that stress shielding progressed differently over time between the two stem types, whereas cancellous condensation and cortical hypertrophy did not show significant stem-dependent differences when within-patient clustering was considered.

Longitudinal mixed-effects analysis demonstrated that stress shielding progressed differently between the two stem designs over time. Although both stems showed increasing stress shielding during follow-up, the increase was significantly greater in the Taper stem. The widening difference observed after 5 years suggests that stem geometry influences long-term load transfer patterns. The more distal corundumized surface of the Taper stem may promote distal fixation and reduce physiological loading of the proximal femur, thereby contributing to progressive proximal bone resorption [30,31].

Over time, this can lead to bone resorption and weakening of the proximal femoral structure, which is clinically relevant, as stress shielding has been associated with an increased risk of periprosthetic fractures, especially in older patients or those with osteoporosis [32,33,34].

In contrast, the Midcoat stem demonstrated more favorable proximal load transfer characteristics, as indicated by lower stress shielding grades and a higher frequency of bone ingrowth fixation. The fluted, grit-blasted distal surface of the Midcoat stem, designed to improve rotational stability without excessive diaphyseal engagement, may preserve the metaphyseal bone stock more effectively. These findings agree with biomechanical principles and support previous reports that emphasize the importance of proximal load sharing in cementless stem design [35,36].

Our results are consistent with those of previous midterm studies involving the VerSys stem family [36,37]. For example, Hamano et al. [37] reported that over a 5–9-year period, grade ≥ 3 stress shielding occurred in 55% of cases with the T stem and approximately 21% with the M stem. In our study, at over 15 years, we observed even higher rates, 64.6% in the T group and 43.4% in the M group, indicating that stress shielding continues to progress over time, particularly in distally fixed designs.

Furthermore, our findings align with the biomechanical studies by Arabnejad et al. and Loha et al., which demonstrated that femoral stems with more distal fixation geometries tended to induce higher stress shielding in the proximal femur [32,34]. These studies, along with current clinical data, underscore the need to optimize stem geometry to balance initial stability with long-term bone preservation.

In contrast, generalized linear mixed modeling demonstrated no significant main effects of stem type or stem × zone interactions on cancellous condensation and cortical hypertrophy. Although zonal variation was observed for cancellous condensation, these patterns were not dependent on stem design after accounting for within-patient correlations. Similarly, cortical hypertrophy did not demonstrate any significant stem-related or zonal differences in the mixed model. These findings suggest that although localized bone responses may occur in specific anatomical regions, they do not appear to differ substantially between the two stem designs when analyzed using an appropriate hierarchical framework.

The present findings suggest that the most important difference between the two stems is the progression of stress shielding over time. Although cancellous condensation and cortical hypertrophy varied among Gruen zones, these changes were not significantly influenced by stem design after statistical adjustment. In contrast, stress shielding showed a clear stem-dependent pattern, indicating that differences in stem geometry primarily affect proximal load transfer and long-term bone preservation.Preservation of the proximal femoral bone stock is critical for the long-term success of THA, particularly in younger and more active patients, who are more likely to require future revision surgeries. For example, recent reviews have noted that younger age at primary THA correlates with a higher lifetime revision risk, and bone-conserving or metaphyseal-anchoring stem designs have been developed to address this issue [38,39]. A clinical series of young adult patients confirmed favorable early outcomes using short bone-conserving stems [38,39]. Excessivestress shielding may not only elevate the risk of periprosthetic femoral fracture by weakening the proximal diaphyseal bone but also create a challenging scenario for revision arthroplasty due to compromised proximal bone stock and altered load-transmission patterns. For instance, proximal bone loss due to stress shielding has been associated with an increased fracture incidence after THA [40,41]. Our study suggests that the Midcoat stem may be more favorable for long-term outcomes by promoting proximal fixation and reducing severe stress shielding.

Although no aseptic loosening was observed in either group, indicating that both stem designs achieved durable long-term fixation, national joint registries have reported that cumulative revision rates continue to increase with longer follow-up periods [42,43]. The higher proportion of fibrous fixation in the Taper group did not lead to clinical failure during the study period, and although differences in the progression of stress shielding were identified, these did not translate into detectable differences in revision risk within this cohort. Nevertheless, progressive proximal bone loss associated with stress shielding may compromise bone stock and increase the complexity of revision surgery when it eventually becomes necessary.This study had several limitations. First, although the follow-up duration was long, this was a retrospective study. Second, clinical outcomes such as pain or functional scores were not available for correlation with radiographic findings. Third, although mixed-effects modeling was used to account for repeated-measures and within-patient clustering, unmeasured confounders may still exist. Fourth, genetic factors that may influence osseointegration, bone remodeling, and long-term implant adaptation were not evaluated. Previous studies have suggested that host genetic background may contribute to variability in THA outcomes. Therefore, some of the observed differences may not be attributable solely to implant design.

Despite these limitations, the present study provides one of the longest longitudinal evaluations of femoral remodeling in the VerSys stem system and demonstrates that stem geometry influences the trajectory of stress shielding over time, whereas zonal bone responses such as cancellous condensation and cortical hypertrophy appear to be less dependent on stem design.

## 5. Conclusions

In this long-term retrospective study evaluating femoral bone remodeling after THA using the VerSys Hip System, longitudinal mixed-effects modeling demonstrated that stress shielding progressed differently between the Taper and Midcoat stems over more than 15 years of follow-up. The Taper stem exhibited a significantly greater increase in stress shielding over time, indicating distinct patterns of load adaptation associated with the stem geometry. In contrast, cancellous condensation and cortical hypertrophy did not show significant stem-dependent differences when analyzed using generalized linear mixed models that accounted for within-patient clustering. These findings suggest that the primary long-term difference between the two stem designs lies in the trajectory of stress shielding progression rather than in zonal bone responses. Although no cases of aseptic loosening were observed, the differences in proximal bone remodeling may have implications for long-term bone preservation. Further prospective studies integrating clinical and biomechanical assessments are warranted to clarify the clinical relevance of these radiographic findings.

## Figures and Tables

**Table 1:** Demographic date of the participants.

		Total (n = 157)	M Group (n = 92)	T Group (n = 65)		*p*-Value
Age (years) ^1^		57.6 ± 9.6	56.9 ± 8.7	57.9 ± 9.4		0.529 ^2^
Sex (Male/Female)		32/125	19/73	13/52		0.921 ^3^
Surgical side (right/left)		85/70	47/45	38/25		0.257 ^3^
Primary disease	HOA	123	72	51	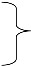	0.990
	ONFH	25	15	10		
	Others	9	5	4		
Approach	DL	110	70	40	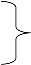	0.014
	ALS	16	4	12		
	OCM	31	18	13		
Radiographic anteversion (°) ^1^		41.0 ± 5.8	40.2 ± 6.5	41.3 ± 5.6		0.282 ^2^
Radiographic inclination (°) ^1^		21.3 ± 6.9	21.4 ± 7.0	21.3 ± 6.6		0.802 ^2^
Stem anteversion (°) ^1^		18.4 ± 12.7	17.6 ± 11.8	20.7 ± 14.5		0.143 ^2^

^1^ mean ± standard deviation. ^2^ *t*-test. ^3^ chi-square test. HOA: hemilateral hip osteoarthritis, ONFH: osteonecrosis of femoral head, DL: direct lateral approach, ALS: anterolateral supine approach, OCM: Orthopädische Chirurgie München approach.

**Table 2:** Fixation of the stem.

	M Group (n = 92)	T Group (n = 65)		*p*-Value
bone ingrowth	83, 90.2%	50, 76.9%	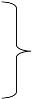	0.074
fibrous stable	9, 9.8%	15, 23.1%		
unstable	0, 0%	0, 0%		

**Table 3:** Comparison of the rate of severe stress shielding.

Survey Period	Midcoat	Taper	*p*-Value
1 year	92/0 *	65/0 *	/
3 year	88/4 *	61/4 *	0.580
5 year	86/6 *	51/14 *	0.004
10 year	81/11 *	35/30 *	<0.001
15 year	73/19 *	27/38 *	<0.001
Final	72/20 *	18/47 *	<0.001

* none, grade 1 and 2/grade 3 and 4.

## Data Availability

The datasets generated and/or analyzed during the current study are not publicly available due to ethical restrictions and the inclusion of personal medical information but are available from the corresponding authors upon reasonable request.

## Abbreviations

The following abbreviations are used in this manuscript:

CC	Cancellous condensation
CH	Cortical hypertrophy
HA/TCP	Hydroxyapatite/tricalcium phosphate
M group	VerSys Midcoat stem
SS	Stress shielding
THA	Total hip arthroplasty
T group	VerSys Taper stem

## References

[1] Maradit Kremers H., Larson D.R., Crowson C.S., Kremers W.K., Washington R.E., Steiner C.A., Jiranek W.A., Berry D.J. (2015). Prevalence of total hip and knee replacement in the United States. J. Bone Jt. Surg. Am..

[2] Fontalis A., Epinette J.A., Thaler M., Zagra L., Khanduja V., Haddad F.S. (2021). Advances and innovations in total hip arthroplasty. SICOT-J..

[3] Foissey C., Fauvernier M., Fary C., Servien E., Lustig S., Batailler C. (2020). Total hip arthroplasty performed by direct anterior approach—Does experience influence the learning curve?. SICOT-J..

[4] Kang J.S., Moon K.H., Park S.R., Choi S.W. (2010). Long-term results of total hip arthroplasty with an extensively porous coated stem in patients younger than 45 years old. Yonsei Med. J..

[5] Özdemir E., Kuijpers M.F.L., Schreurs B.W., Rijnen W.H.C. (2023). Long-term follow-up of 96 patients younger than age 25 with 119 primary cemented total hip arthroplasties. Acta Orthop..

[6] Imai N., Miyasaka D., Ibuchi S., Kimura K., Hirano Y., Horigome Y., Kawashima H. (2024). The long-term efficacy of computed tomography-navigated total hip arthroplasty: An 18-year follow-up study. J. Clin. Med..

[7] Kim Y.H., Park J.W., Jang Y.S. (2021). Long-Term Survival (up to 34 years) of Retained cementless Anatomic Femoral Stem in Patients <50 years old. J. Arthroplast..

[8] Zang J., Uchiyama K., Moriya M., Li Z., Fukushima K., Yamamoto T., Takahira N., Takaso M., Liu J., Feng W. (2018). Long-term clinical and radiographic results of the cementless Spotorno stem in Japanese patients: A more than 15-year follow-up. J. Orthop. Surg..

[9] Raffa M.L., Nguyen V.H., Hernigou P., Flouzat-Lachaniette C.H., Haiat G. (2021). Stress shielding at the bone–implant interface: Influence of surface roughness and of the bone-implant contact ratio. J. Orthop. Res..

[10] Guo L., Ataollah Naghavi S.A., Wang Z., Nath Varma S., Han Z., Yao Z., Wang L., Wang L., Liu C. (2022). On the design evolution of hip implants: A review. Mater. Des..

[11] Vajapey S.P., Shah V.M., Li M., Estok D.M. (2025). Cementless fixation in total joint arthroplasty: Factors impacting osseointegration. J. Clin. Orthop. Trauma..

[12] Kim Y.H. (2021). Ultra-short bone conserving cementless femoral stem. Hip Pelvis.

[13] Bonin N., Gedouin J.E., Pibarot V., Bejui-Hughues J., Bothorel H., Saffarini M., Batailler C. (2017). Proximal femoral anatomy and collared stems in hip arthroplasty: Is a single collar size sufficient?. J. Exp. Orthop..

[14] Bahk J.H., Han S.B., Rhyu K.H., Yoo J.J., Lim S.J., Park K.K., Kim S.M., Lim Y.W. (2024). Identification of essential features in developing a novel femoral stem reflecting anatomical features of East Asian population: A morphological study. J. Clin. Med..

[15] Su Y., Cockerill I., Zheng Y., Tang L., Qin Y.X., Zhu D. (2019). Biofunctionalization of metallic implants by calcium phosphate coatings. Bioact. Mater..

[16] Goyenvalle E., Guyen N.J.M., Aguado E., Passuti N., Daculsi G. (2003). Bilayered calcium phosphate coating to promote osseointegration of a femoral stem prosthesis. J. Mater. Sci. Mater. Med..

[17] Yoon K.S., Kim H.J., Lee J.H., Kang S.B., Seong N.H., Koo K.H. (2007). A randomized clinical trial of cementless femoral stems with and without hydroxyapatite/tricalcium-phosphate coating: An 8- to 12-year follow-up study. J. Arthroplast..

[18] Ibuchi S., Imai N., Horigome Y., Hirano Y., Kimura K., Kawashima H. (2024). Long-term outcomes and a radiological assessment of hydroxyapatite-tricalcium phosphate-coated total hip arthroplasty (trilogy/Zimmer): A long-term follow-up study. Medicina.

[19] Chambers B., St Clair S.F.S.T., Froimson M.I. (2007). Hydroxyapatite-coated tapered cementless femoral components in total hip arthroplasty. J. Arthroplast..

[20] Bourne R.B., Rorabeck C.H., Patterson J.J., Guerin J. (2001). Tapered titanium cementless total hip replacements: A 10- to 13-year followup study. Clin. Orthop. Relat. Res..

[21] Kendrick J.B., Noble P.C., Tullos H.S. (1995). Distal stem design and the torsional stability of cementless femoral stems. J. Arthroplast..

[22] Steinbrück A., Grimberg A.W., Elliott J., Melsheimer O., Jansson V. (2021). Short versus conventional stem in cementless total hip arthroplasty: An evidence-based approach with registry data of mid-term survival. Orthopade.

[23] Kaku N., Pramudita J.A., Yamamoto K., Hosoyama T., Tsumura H. (2022). Stress distributions of the short stem and the tapered wedge stem at different alignments: A finite element analysis study. J. Orthop. Surg. Res..

[24] Tamaki T., Jonishi K., Miura Y., Oinuma K., Shiratsuchi H. (2018). Cementless tapered-wedge stem length affects the risk of periprosthetic femoral fractures in direct anterior total hip arthroplasty. J. Arthroplast..

[25] Kawaji H., Uematsu T., Hoshikawa N., Watanabe H., Takai S. (2016). Mid-term clinical results of VerSys Hip System (Zimmer) uncemented total hip replacement arthroplasty. J. Nippon. Med. Sch..

[26] González Della Valle A., Comba F., Zoppi A., Salvati E.A. (2006). Favourable mid-term results of the VerSys CT polished cemented femoral stem for total hip arthroplasty. Int. Orthop..

[27] Engh C.A., Bobyn J.D., Glassman A.H. (1987). Porous-coated hip replacement. The factors governing bone ingrowth, stress shielding, and clinical results. J. Bone Jt. Surg. Br..

[28] Gruen T.A., McNeice G.M., Amstutz H.C. (1979). “Modes of failure” of cemented stem-type femoral components: A radiographic analysis of loosening. Clin. Orthop. Relat. Res..

[29] Engh C.A., Massin P., Suthers K.E. (1990). Roentgenographic assessment of the biologic fixation of porous-surfaced femoral components. Clin. Orthop. Relat. Res..

[30] Scheerlinck T., Casteleyn P.P. (2006). The Design features of cemented femoral hip implants. J. Bone Jt. Surg. Br..

[31] Kim Y.H., Park J.W., Kim J.S., Kang J.S. (2014). Long-term results and bone remodeling after THA with a short, metaphyseal-fitting anatomic cementless stem. Clin. Orthop. Relat. Res..

[32] Loha T., Bhattacharya R., Pal B., Amis A.A. (2024). A novel design of hip-stem with reduced strain-shielding. Proc. Inst. Mech. Eng. Part H J. Eng. Med..

[33] Naghavi S.A., Tamaddon M., Garcia-Souto P., Moazen M., Taylor S., Hua J., Liu C. (2023). A novel hybrid design and modelling of a customised graded Ti-6Al-4V porous hip implant to reduce stress-shielding: An experimental and numerical analysis. Front. Bioeng. Biotechnol..

[34] Arabnejad S., Johnston B., Tanzer M., Pasini D. (2017). Fully porous 3D printed titanium femoral stem to reduce stress-shielding following total hip arthroplasty. J. Orthop. Res..

[35] Khanuja H.S., Vakil J.J., Goddard M.S., Mont M.A. (2011). Cementless femoral fixation in total hip arthroplasty. J. Bone Jt. Surg. Am..

[36] Klein G.R., Levine H.B., Nafash S.C., Lamothe H.C., Hartzband M.A. (2009). Total hip arthroplasty with a collarless, tapered, fiber metal proximally coated femoral stem: Minimum 5-year follow-up. J. Arthroplast..

[37] Hamano D., Hagio K., Koyama T., Saito M. (2012). Mid-term results of VerSys FMT stem. Cent. Jpn. J. Orthop. Trauma. Surg..

[38] Anderl C., Steinmair M., Hochreiter J. (2022). Bone preservation in total hip arthroplasty. J. Arthroplast..

[39] Zhen P., Chang Y., Yue H., Chen H., Zhou S., Liu J., He X. (2021). Primary total hip arthroplasty using a short bone-conserving stem in young adult osteoporotic patients with Dorr type C femoral bone. J. Orthop. Surg. Res..

[40] Savio D., Bagno A. (2022). When the total hip replacement fails: A review on the stress-shielding effect. Processes.

[41] Burchard R., Graw J.A., Soost C., Schmitt J. (2023). Stress shielding effect after total hip arthroplasty varies between combinations of stem design and stiffness-a comparing biomechanical finite element analysis. Int. Orthop..

[42] Australian Orthopaedic Association National Joint Replacement Registry Hip, Knee and Shoulder Arthroplasty, 2025. [Annual Report]. https://aoanjrr.sahmri.com/annual-reports-2025.

[43] National Joint Registry [22nd Annual Report]. Hips, 2025. https://reports.njrcentre.org.uk/downloads.

